# Patient-reported outcome measures for monitoring primary care patients with depression: PROMDEP feasibility randomised trial

**DOI:** 10.1136/bmjopen-2016-015266

**Published:** 2017-03-30

**Authors:** Tony Kendrick, Beth Stuart, Geraldine M Leydon, Adam W A Geraghty, Lily Yao, Rachel Ryves, Samantha Williams, Shihua Zhu, Christopher Dowrick, Glyn Lewis, Michael Moore

**Affiliations:** 1 Primary Care and Population Sciences, University of Southampton, Southampton, UK; 2 Institute of Psychology Health and Society, University of Liverpool, Liverpool, UK; 3 Institute of Epidemiology and Health Care, University College London, London, UK

**Keywords:** depression, PRIMARY CARE, Patient reported outcome measures

## Abstract

**Objectives:**

To determine the feasibility of a trial of patient-reported outcome measures (PROMs) for monitoring primary care patients with depression.

**Design:**

Partly individually randomised, partly cluster-randomised controlled trial.

**Setting:**

Nine general practices in Southern England.

**Participants:**

47 adults with new episodes of depression: 22 intervention, 25 control.

**Randomisation:**

Remote computerised sequence generation and allocation.

**Interventions:**

Patient Health Questionnaire, Distress Thermometer Analogue Scale and PSYCHLOPS problem profile for monitoring depression, following diagnosis and at 10–35 days later. Feedback of scores to patients was determined by practitioners.

**Blinding:**

Non-blinded, using self-completed measures.

**Primary outcome:**

Beck Depression Inventory (BDI-II).

**Secondary outcome measures:**

Work and Social Adjustment Scale (WSAS), EuroQol Five-item, Five-level (EQ-5D-5L) Scale for quality of life, modified Client Service Receipt Inventory for costs, Medical Informant Satisfaction Scale (MISS), qualitative interviews with 14 patients and 13 practice staff about feasibility and acceptability of trial design.

**Results:**

Three practices failed to recruit the target of six patients in 12 months. Follow-up rates were intervention patients: 18 (82%) at 12 weeks and 15 (68%) at 26 weeks; controls: 18 (72%) and 15 (60%), respectively. At 12 weeks, mean BDI-II score was lower among intervention group patients than controls by 5.8 points (95% CI −11.1 to −0.5), adjusted for baseline differences and clustering. WSAS scores were not significantly different. At 26 weeks, there were no significant differences in symptoms, social functioning, quality of life or costs, but mean satisfaction score was higher among controls by 22.0 points (95% CI −40.7 to −3.29). Intervention patients liked completing PROMs, but were disappointed when practitioners did not use the results to inform management.

**Conclusions:**

PROMs may improve depression outcome in the short term, even if PROM scores do not inform practitioners' management. Challenges in recruiting and following up patients need addressing for a definitive trial of relatively brief measures which can potentially inform management. https://www.isrctn.com/search?q=97492541

**Trial registration number:**

ISRCTN 97492541; Pre-results.

**Strengths and limitations of this study**

- Pragmatic trial with few exclusion criteria, readily generalisable.
- Patients were randomly allocated with concealment of allocation from patients until after informed consent had been obtained and baseline measures completed.
- Patients, practitioners and assessors could not be blinded to allocation.
- Self-report research outcome measures should have prevented observer rating bias.
- Despite the small sample size, we did find a difference in the primary outcome.

## Background

Depression is common and costly. The estimated prevalence among adults in the UK is 11.1%, including major depressive disorder in 3.3% and mixed depression and anxiety in 7.8%.^1^ It can lead to chronic disability, poor quality of life, suicide in some cases and high levels of health service use and economic costs. The King's Fund have estimated that 1.45 million people will have depression in England by 2026, and total societal costs will be £12.2 billion per year, including healthcare, social services and lost employment.^2^

The National Institute for Health and Care Excellence (NICE) depression guidelines recommend different interventions for moderate to severe depression than for mild depression.^3^ However, general practitioner (GP) clinical assessments of the severity of depression vary and are often inaccurate when compared with validated measures.^4^ ^5^ Consequently, some GPs do not accurately target treatment to patients most likely to benefit,^6–8^ reducing the cost-effectiveness of treatment, which needs to be optimised given the impact of depression.

As a result of these findings, NICE recommends that health professionals consider using validated questionnaire measures of severity at diagnosis to help target treatment.^3^ Between 2006 and 2013, the UK GP contract Quality and Outcomes Framework (QOF) paid GPs to use symptom questionnaires as part of their assessments of depression severity at the outset of treatment for patients with a new diagnosis.^9^ Symptom questionnaire assessments at follow-up of treated patients were also incentivised through the QOF between 2009 and 2013, to promote follow-up reassessment.^10^

Some patients value using symptom questionnaires to assess treatment effectiveness and monitor their progress,^11^ ^12^ and some GPs also value them for monitoring patients' progress. The likelihood of antidepressant treatment and/or referral for psychological therapy is significantly associated with higher symptom questionnaire scores at diagnosis,^13^ and decisions to change treatment are significantly associated with changes in scores at follow-up.^14^

However, the use of symptom questionnaires is disliked by some GPs, who worry they intrude in sensitive consultations and undermine professional autonomy and, doubting their validity, prefer using clinical judgement to assess severity and response to treatment.^11^ ^15^ In 2012, a NICE-commissioned systematic review concluded that the evidence supporting questionnaires was not strong enough to require their use in QOF depression indicators.^16^ Current QOF guidance suggests formal assessment questionnaires can be used to measure severity at reviews 10–56 days after diagnosis, but this is optional rather than required to receive payments for the reviews.^17^

The QOF depression symptom questionnaires are an example of patient-reported outcome measures (PROMs), the use of which has been promoted in recent years to increase patient involvement in their own care.^18^ A recent Cochrane systematic review of the use of PROMs in the treatment of common mental health disorders (CMHDs) including depression found some evidence of benefit for patients identified as having a lack of improvement early on in treatment, but the research was generally of low quality.^19^ More research is required, particularly in primary care where most CMHDs are treated.

If using symptom questionnaires and other PROMs is beneficial even to a modest extent, they are likely to be cost-effective given their low cost, and the benefits at a population level would be considerable in public health terms, given the high cost to the nation of depression. Randomised trials of using PROMs to monitor patients' progress in primary care are however needed to inform practitioners definitively whether their use is beneficial, given their justifiable doubts about the validity of the approach.

We decided a feasibility study was needed first, to determine whether practices in England would agree to use PROMs with patients during consultations for the assessment of depression at diagnosis and follow-up. It was also needed to determine whether a trial randomised at patient level would be preferable to cluster-randomising whole practices, which might need a bigger sample size, depending on the intracluster correlation coefficient (ICC) between practices, which could also be estimated through a feasibility trial.

## Aim

To test the feasibility of conducting a randomised controlled of PROMs for monitoring outcomes for patients with depression in primary care.

### Objectives

- To determine key elements of the best design for a trial, including:
  1. The willingness and ability of general practices to:
    - recruit patients during consultations at which depression is diagnosed
    - recruit through mail-outs to patients recorded as having consulted for depression
    - be randomised to intervention or control arms as whole practices (cluster design)
    - have patients individually randomised to intervention or control arms
  2. The willingness of patients with depression to:
    - complete PROMs in the intervention arm
    - complete measures of symptoms, functioning, quality of life and service use
  3. Numbers of eligible patients found per practice
  4. Rates of recruitment and follow-up
- To test the feasibility and acceptability of administering the Patient Health Questionnaire (PHQ-9)^20^ for depressive symptoms, Distress Thermometer Analogue Scale^21^ and PSYCHLOPS individual problem profile^22^ as PROMs for depression
- To explore effects of the intervention on depressive symptoms, social functioning, quality of life, satisfaction and costs
- To estimate the ICC for the primary outcome to use in calculating the necessary increase in sample size for a cluster-randomised full trial.

## Methods

### Trial design

Parallel group, partly individually randomised, partly cluster-randomised trial, with 1:1 allocation between intervention and control arms.

### Participants

Group general practices in and around Southampton, Southern England, were recruited through the National Institute for Health Research (NIHR) Clinical Research Network (CRN).

Eligibility criteria were adult patients aged 18 years and above, diagnosed with a new episode of depression. Exclusion criteria were previous treatment for depression within 12 months, comorbid dementia, psychosis, substance misuse or serious suicidal ideation needing urgent specialist referral. The diagnosis of a new episode of depression and previous treatment for depression were defined by the participating GPs rather than assessed independently, in keeping with the pragmatic nature of the trial.

Where possible, patients who had been diagnosed with a new episode of depression were recruited opportunistically during consultations by GPs and practice nurses (PNs) and referred to the study team to discuss taking part. Newly diagnosed patients were also identified through medical record searches, designed to be weekly, by practice administrative staff, mailed information about the study and asked if they wished to discuss taking part. Records were searched for 116 Read codes^23^ for depressive diagnoses and symptoms (see online supplementary appendix 1 for a list of specific Read codes).

10.1136/bmjopen-2016-015266.supp1 supplementary appendix

### Intervention

The intervention was the administration of the three PROMs, the PHQ-9^20^ for depressive symptoms, the Distress Thermometer Analogue Scale for distress^21^ and the PSYCHLOPS profile rating of one or two problems individual to the patient,^22^ administered as soon as possible after diagnosis, reviewed by the GP or PN and repeated at a follow-up GP or PN consultation 10–35 days later. Table 1 shows what each of the PROMs measures, and the rationale for their inclusion.

**Table 1:** Study patient-reported outcome measures (PROMs)

Measure	What it measures	Rationale for inclusion
Patient Health Questionnaire, nine-item version (PHQ-9)^20^	Severity of depression using nine questions covering diagnostic criteria for major depression. Total scores are categorised as minimal (1–4), mild (5–9), moderate (10–14), moderately severe (15–19) and severe (20–27).	Validated in UK primary care^24^ and the most commonly used symptom questionnaire in UK general practice when incentivised through the Quality and Outcomes Framework.^13^
Distress thermometer single-item question screen originally developed for people with cancer^21^ but can measure distress coming from any source	Visual Analogue Scale on which patients indicate how distressed they have been during the past week on a scale of 0–10. Scores of 4 or more indicate a significant level of distress that should be investigated further.	A rapid indication of change in distress level. Does not require English skills to complete, unlike questionnaires.
PSYCHLOPS psychological outcomes profile,^22^ a one-page three-item self-report measure	Patient descriptions of their own particular individual problem or two problems, their ratings (0–5) of how their problem(s) affect their daily functioning, and their ratings (0–5) of overall well-being.	Approved by the Plain English Campaign and carries the ‘Crystal Mark’ for clarity. Shown to be highly sensitive to change during the course of psychotherapeutic interventions.^22^

Researchers visited patients willing to be contacted, either at home or at their practices, sought informed written consent, carried out baseline assessments and administered the three PROMs to intervention group patients, with a brief explanation of each measure and further discussion of any questions on the PROMs patients were unsure about. Patients completed the PROMs on paper and were asked to book an appointment with their GP or PN within a week, or as soon as possible, and to take the completed measures along, to discuss the results. Patients were not routinely given feedback on the meaning of the PROM scores by the researcher: routine feedback of results was left to the participating practitioners. If patients asked for immediate feedback, the researchers informed them only what their PROM scores were in relation to the possible maximum scores, and advised them to speak to their GP/PN for further information and guidance.

Participating GPs and PNs were given up to half an hour's instruction on the meaning of the scores on the three PROMs at the start of the study. They were asked to take the PROM scores into account at their consultations with participating patients within days of completion of the first set of PROMs. They were also asked to provide the patients with another set of the three PROMs to complete again immediately prior to follow-up consultations 10–35 days later. Advice was given on the meaning of scores on the PROMs at the consultation following diagnosis and of changes in scores between the first and second follow-up consultations. See online supplementary appendix 2 for the advice given about the meaning of PROM scores.

10.1136/bmjopen-2016-015266.supp2 supplementary appendix

Whether or not feedback on the results of the PROMs was given by the practitioners to the patients, and any treatment and further follow-up provided for depression, was left to the discretion of participating practitioners for patients in intervention and control groups.

Control group patients did not complete any PROMs. All patients completed the research outcome measures (below) but were not given feedback on the results of those assessments (see online supplementary appendix 3 for an overview of study intervention and control procedures).

10.1136/bmjopen-2016-015266.supp3 supplementary appendix

### Assessments

Patients were recruited over a 12-month period and followed up for 26 weeks each, with assessments at baseline, 12 and 26 weeks follow-up. Baseline measures included sociodemographic details (age, gender, length of education, employment, cohabitation), duration of symptoms, previous history of depression, previous treatment and the Generalised Anxiety Disorder (GAD-7) Questionnaire for anxiety symptoms.^25^

### Outcomes

The primary outcome measured at 12 and 26 weeks follow-up was depressive symptoms on the Beck Depression Inventory 2nd edition (BDI-II).^26^ Social functioning on the Work and Social Adjustment Scale (WSAS)^27^ and quality of life on the EuroQol Five-item, Five-level (EQ-5D-5L) Scale^28^ were also measured at 12 and 26 weeks follow-up. At 26 weeks follow-up, use of services was determined using a modified version of the Client Services Receipt Inventory^29^ to allow calculation of National Health Service (NHS) service costs, and patient satisfaction was determined using the Medical Informant Satisfaction Scale (MISS).^30^

### Sample size

A formal sample size calculation was not performed, as the aim was to explore effectiveness not to determine it accurately. We aimed to obtain primary outcome data on 40 patients, 20 in each arm, which we judged would be sufficient to allow estimation of rates of recruitment and follow-up, and of the variance in the primary outcome measure the BDI-II, together with its ICC between practices, to inform a sample size calculation for the main trial if a cluster-randomised design were to be chosen.

We estimated an average group practice could recruit six patients in 12 months. This was based on the mean number of patients per practice of 23 per year with a new episode of depression found to have been assessed using the QOF-incentivised PHQ-9 in a previous observational study^14^ and an assumption that around 25% of diagnosed patients would consent to participate. (The prevalence of depression is considerably higher,^1^ but we aimed to recruit only patients with an incident episode rather than all patients currently suffering from depression.) We further anticipated 15% would drop out of follow-up based on a previous trial of antidepressants^31^ in primary care which meant we would need to recruit 48 patients from eight practices to obtain primary outcome data on around 40 patients at follow-up.

### Randomisation

Four of the practices were cluster randomised to intervention or control arms, while in the remaining practices patients were individually randomised, in order to explore the feasibility and acceptability of both methods. Randomisation was carried by the study statistician (BS) using computerised sequence generation. The researchers were aware of randomisation status for patients of cluster-randomised practices. For individually randomised patients, researchers telephoned the statistician for allocation to intervention or control after obtaining informed consent and carrying out baseline assessments.

### Blinding

Patients, practitioners and researchers could not be blinded to allocation given the nature of the intervention. Self-report outcome measures were used to prevent observer rating bias.

### Analysis

Feasibility and acceptability were assessed through analysis of rates of recruitment, dropout and follow-up. Patients rated the ease of completion of the measures and time taken using 5-point Likert scales.

Differences at 12 and 26 weeks follow-up between intervention and control patients in depressive symptoms and social functioning were explored using a linear mixed model adjusting for sociodemographic characteristics, baseline depressive and anxiety symptoms, and for clustering by practice by including practice as a random effect. Patient satisfaction, quality of life (in quality adjusted life years (QALYs)) and costs over 26 weeks were also compared between arms. The analysis included only patients for whom we had outcome data (ie, complete cases).

The acceptability of trial procedures and chosen PROMs was also explored through semistructured qualitative interviews with samples of participating patients and health professionals, aiming to interview 15–20 of each. Interviews were transcribed verbatim and analysed using an inductive thematic analysis approach.^32^

The study was sponsored by the University of Southampton.

## Results

### Recruitment

Recruitment of practices and patients took place between September 2014 and February 2016 inclusively, and the 26-week follow-ups ended in September 2016. Eight practices were recruited to the study in the first month as planned. Four of them were cluster randomised to intervention or control arms, and in the remaining practices, patients were individually randomised. However, two of the original sample of practices failed to recruit any patients within the first 3 months. We therefore replaced these practices with two which recruited patients more quickly, but due to slower than desired recruitment among some of the other participating practices, we also agreed an amendment to our protocol with the Research Ethics Committee (REC) to recruit from a ninth practice, in order eventually to achieve recruitment of 47 of our target of 48 patients. One of the three replacement practices was cluster randomised to replace a cluster-randomised practice which had dropped out, and in the other two, patients were individually randomised.

Three practices recruited six patients within 8 weeks, while three failed to recruit six in a year, and three were intermediate recruiters. Two of the three practices that failed to recruit had individual mitigating circumstances. One was recruited to the study late, with only a 1-month time frame, so did well to recruit three patients. Another decided to stop actively taking part in all research and did not continue to recruit patients. Feedback from the third practice suggested that greater clarity on study eligibility criteria and guidance to promote the study during consultations was needed to prompt recruitment.

**Figure 1:**
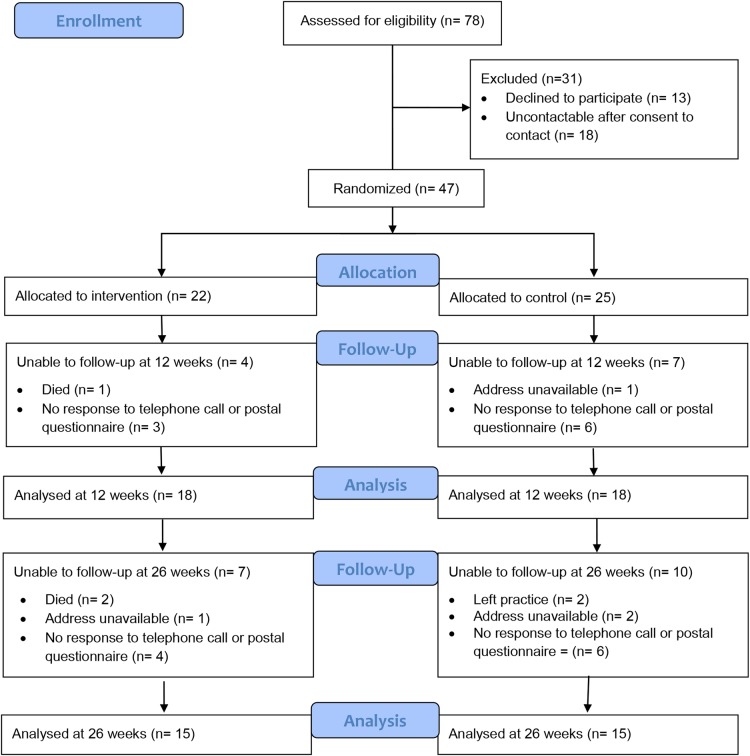
CONSORT flow diagram of patient participation.

From the nine practices, a total of 78 patients agreed to discuss participation, of whom 47 (60%) were randomised (37 (79%) recruited in consultations and 10 (21%) through mail-outs). Practice logs showed that fewer than 10% of patients identified as eligible and mailed information about the study returned reply slips indicating whether or not they were interested in participating. Of the 31 patients (40%) who were not recruited, 18 (23%) were uncontactable at baseline and 13 (17%) declined after initial contact. The main reasons for declining were no longer being interested in taking part or having competing commitments (see figure 1, consensus statement on reporting randomised trials (CONSORT) flow diagram of patient participation).

### Baseline characteristics

Of the 47 recruited patients, 29 (62%) were women, 46 (98%) were white and 31 (66%) were in employment. The average age was 44 years and average age of leaving education 19. Study arms were reasonably well balanced at baseline (table 2), except more intervention group patients were married or cohabiting, and control patients' scores for depression, social functioning and anxiety were all slightly worse on average and less variable.

**Table 2:** Baseline characteristics

Characteristic	Control patients (n=25)	Intervention patients (n=22)
Female	16 (64.0%)	13 (59.0%)
Age (mean (SD))	43.1 (17.1)	44.7 (18.5)
White ethnic group	24 (96.0%)	22 (100%)
Marital status		
Married/cohabiting	6 (24.0%)	12 (54.5%)
Widowed/separated/divorced	8 (32.0%)	6 (27.3%)
Single	11 (44.0%)	4 (18.2%)
Any dependents at home	9 (36.0%)	7 (31.8%)
Age left education	18.6 (5.3)	18.8 (3.4)
Economic position		
Full-time/part-time work	17 (68.0%)	14 (63.6%)
Sick/disabled	2 (8.0%)	1 (4.6%)
Unemployed	1 (4.0%)	1 (4.6%)
Retired/student/homemaker	5 (20.0%)	5 (22.7%)
Other	0	1 (4.6%)
BDI-II total score, mean (SD)	26.92 (7.93)	23.90 (11.92)
WSAS total score, mean (SD)	21.88 (9.37)	18.13 (10.00)
GAD-7 total score, mean (SD)	14.32 (5.27)	11.64 (5.83)

BDI-II, Beck Depression Inventory, 2nd edition;^24^ GAD-7, Generalised Anxiety Disorder Scale;^23^ WSAS, Work and Social Adjustment Scale.^25^

### Follow-up rates

At 12 weeks, 18 of 22 intervention arm patients (82%) and 18 of 25 controls (72%) completed the outcome measures, and at 26 weeks, 15 (68%) and 15 (60%), respectively (see CONSORT diagram, figure 1). Of those followed up, 29 patients (81%) completed questionnaires face-to-face at 12 weeks and 26 (87%) at 26 weeks, the rest completing them only after further follow-up by post (19% at 12 weeks and 13% at 26). No patients proved contactable to complete outcome measures over the telephone.

### Outcomes

Outcome scores at baseline, 12 and 26 weeks follow-up are shown in table 3. At 12 weeks, the intervention group adjusted mean score for depressive symptoms on the BDI-II was significantly lower than the control group by 5.8 points (95% CI −11.1 to −0.5) after adjusting for baseline depression scores, anxiety, sociodemographics, psychotropic medication use and clustering by practice. Adjusted mean score on the WSAS at 12 weeks was lower (better) in the intervention group than the controls by a mean of 3.0 points, which was not statistically significant (95% CI −7.3 to 1.3).

**Table 3:** Outcome measures at baseline and follow-up

		Control group			Intervention group		
Measures		Baseline (n=25)	12 weeks (n=18)	26 weeks (n=15)	Baseline (n=22)	12 weeks (n=18)	26 weeks (n=15)
Depression (BDI-II)	Mean (SD)	26.92 (7.93)	19.22 (11.62)	15.53 (10.04)	23.90 (11.92)	12.00 (8.93)	14.13 (12.54)
Social functioning (WSAS)	Mean (SD)	21.88 (9.37)	14.89 (9.30)	14.93 (10.79)	18.13 (10.00)	10.94 (8.12)	12.07 (11.35)
Anxiety (GAD-7)	Mean (SD)	14.32 (5.27)	–	–	11.64 (5.83)	–	–
Quality of life (EQ-5D-5L)	Mean (SD)	0.624 (0.284)	0.698 (0.246)	0.674 (0.299)	0.633 (0.242)	0.759 (0.105)	0.764 (0.158)
Satisfaction (MISS)	Mean (SD)	–	–	148.93 (34.19)	–	–	137.93 (34.74)

BDI-II, Beck Depression Inventory, 2nd edition;^24^ EQ-5D-5L, EuroQol Quality of Life Scale;^26^ GAD-7, Generalised Anxiety Disorder Scale;^23^ MISS, Medical Informant Satisfaction Scale;^28^ WSAS, Work and Social Adjustment Scale.^25^

At 26 weeks, there were no significant differences between the groups in symptoms or social functioning (adjusted mean BDI-II was slightly worse in intervention arm by 2.5 points (95% CI −1.7 to 6.7); adjusted mean WSAS was lower by 0.3 points (95% CI −5.2 to 4.6)). However, the adjusted mean MISS satisfaction score was 22.0 points higher in the control group (95% CI −40.7 to −3.29).

Baseline EQ-5D-5L quality of life scores were similar among intervention and control group patients (table 3). Scores were improved at 12 weeks for both groups although slightly higher among intervention patients than controls, and scores went down again at 26 weeks among controls.

The mean QALY gain over 26 weeks was 0.382 (SD 0.046) for intervention patients and 0.336 (0.132) for controls, giving a non-significant difference of 0.047 (95% CI −0.036 to 0.129). Mean depression-related NHS service costs per patient over 26 weeks were similar: control arm £216 (95% CI £135, £297), intervention arm £231 (£129, £332), including £16 per patient for an estimated 5 min GPs or PNs spent dealing with PROM results.

### Intracluster correlation coefficients

There was no evidence in this sample of clustering by practice for the BDI-II or WSAS: the ICC was zero at baseline for both. After controlling for baseline and randomisation group, the ICC for the BDI-II at 12 weeks was 0.03.

### Ease of completion of outcome measures

On average, participants rated the BDI-II, WSAS and GAD-7 as easy to use and the time taken was under 5 min for each. Ease of completion scores: mean (SD) where 1=not at all easy and 5=very easy: BDI-II 4.29 (0.94), WSAS 4.38 (0.88) and GAD-7 4.38 (0.83). Time taken (minutes): median (IQR): BDI-II 4 (3,5); WSAS 1.5 (1,3); and GAD-7 2 (1,2).

### Qualitative interviews with patients and practice staff

The full qualitative analysis and illustrative quotes from participants will be published separately. We present a brief summary only in this paper.

Fourteen patients were interviewed. Overall, in relation to the feasibility of the study, patients were happy to be randomised (even when randomised to the control arm), were supportive of the use of PROMS (seeing potential benefits for understanding their illness) and reported them relatively easy and quick to complete. There were some difficulties found in discussing the results of PROMS with their practitioners which would need attention in a definitive trial. Some were unable to see the same practitioner for follow-up, and some expressed disappointment at not having feedback on the PROM scores from participating practitioners. Some would have liked a record of changes in scores over time to show their progress.

Interviews were carried out with 10 GPs, one PN and two practice managers. In relation to feasibility practitioners overall considered the use of PROMS to be feasible. Positive feedback on using PROMs included: help with communication, encouraging patients to feed back on symptoms, feel listened to and taken seriously (particularly the PSYCHLOPS); help with treatment planning, confirming decisions, and measuring progress; providing structure in a consultation which could save time; and not missing anything (especially the PHQ-9). Negative feedback included: PROMS are too simplistic (especially the Distress Thermometer), could be difficult for patients to complete (due to insufficient health literacy, sensitive questions or wanting to give the ‘correct’ answer), take time to complete in consultations and may depersonalise interactions. Important areas that would need to be improved to smooth their use in practice included further clarification of patient inclusion criteria, choosing measures that are easy for patients to complete, and more guidance on what to do with the PROM results once completed.

### Methodological changes

Several changes were needed to overcome difficulties in recruiting and following up patients.

As response rates to the practice mail-outs were lower than 10%, we obtained ethics approval to send a revised patient information leaflet which used varied font sizes and coloured text to be more eye-catching, and for PNs to telephone non-responding participants 2 weeks after mail-outs to follow them up more actively (this did not apply to those who had responded to say they were not interested, only those who had not responded at all). However, in the event, the telephone calls did not yield any more participants.

The follow-up research assessments were originally intended to be completed face to face, but we obtained ethics approval to send the research assessment questionnaires by post if patients failed to attend follow-up after two requests. We also obtained approval to send patients a £10 high street shopping gift voucher with the follow-up questionnaires sent by post. These changes between them helped improve follow-up rates by around 10%.

Another change was approved to facilitate active follow-up of non-responding patients. If they did not complete follow-up questionnaires in person or by post, the study team was permitted to try to contact them and complete the primary outcome measure (BDI-II), and two other key outcome measures (WSAS and EQ-5D-5L), over the telephone. However, in the event, no non-responders could be reached by telephone.

Finally, approval was given for an additional practice administrative staff review of the medical records of recruited participants at the end of their participation, as we were able to gather only limited information on service use through the patient questionnaires. These record reviews provided extra information on prescribed medication, number of visits to GPs, PNs and community-based staff, secondary care contacts, hospital admissions and length of hospitalisation where appropriate.

## Discussion

### Principal findings

It is feasible to carry out a randomised trial of PROMs for the assessment and follow-up of depression in primary care in England, although recruitment rates and follow-up rates, particularly in the control arm, would need improving significantly for a larger, definitive trial. Intervention arm patients were happy to complete the PROMs and research outcome questionnaires and valued seeing the results. Differences between arms suggest PROMs may reduce depressive symptoms, yet also reduce patient satisfaction, perhaps because GPs appeared not to value using PROM results to influence management.

### Strengths and limitations

The trial was pragmatic, with few exclusion criteria, and readily generalisable to UK primary care, although the small number of patients recruited in some of the practices raises the question of how representative the sample was of all patients with new episodes of depression. Practices were able to recruit patients in GP/PN consultations and through staff mail-outs to patients, but two practices had to be dropped because of non-recruitment after several months, and difficulties in recruitment in other practices meant we recruited only 47 of the target of 48 patients.

Patients were randomly allocated to intervention or control, with concealment of allocation from patients until after informed consent had been obtained and baseline measures had been completed. However patients, practitioners and assessors could not be blinded to allocation during the trial given the nature of the intervention, although the use of self-report research outcome measures should have prevented observer rating bias.

The study was necessarily small in keeping with testing feasibility, but in spite of this, we did find a difference in the primary outcome measure between arms at 12 weeks follow-up, favouring the intervention. The adjusted difference between arms at 12 weeks as a percentage of the score in the control group was 5.8/19.22=30.1%, which is greater than the minimal clinically important difference (MCID) of a 17.5% reduction in scores from baseline found to correspond to patients' global reports of significant improvement.^33^ We did not determine how many practitioners actually gave feedback on the PROMs to their patients, but our patient interviews suggest not all practitioners did. This might have influenced the clinical outcome of the study, yet some benefit was identified nevertheless.

### Comparison with other studies

The results are in keeping with a US primary care-based controlled trial of feeding back PHQ-9 scores to family practitioners at diagnosis and follow-up, which demonstrated significantly improved patient outcomes over 6 months.^34^ The difference in outcome could not be explained in terms of any significant differences in management, but the benefits of feeding back scores seemed to arise from increasing patients' awareness of their symptoms and their ability to report relevant changes.^35^ That may explain why our patients may have derived benefit from using PROMs even when their GPs did not seem to use the results to inform their care.

### Implications for clinicians, policymakers and research

The implications are mainly for the design of a definitive trial rather than for practice at this stage, although clinicians, policymakers and research funders might be persuaded of the need for a more definitive trial on the basis that short-term differences in outcome favouring the use of PROMs were identified even in this small sample.

To facilitate recruitment, and ensure as representative a sample of patients as possible, a definitive trial should aim to recruit more patients per practice from a smaller number of more committed practices, rather than fewer patients each from a larger number of practices. Depressed patients are often viewed as in need of protection by GPs, who may feel introducing research is intrusive.^36^ A lack of skills in introducing research could be addressed through more training in a smaller group of practices.

Follow-up at 12 weeks of 82% was sufficient in the intervention arm, but needs to be improved from 72% in the control arm, and follow-up at 26 weeks needs to be improved from 68% and 60%, respectively, through taking steps to maintain better contact with patients, obtaining mobile phone numbers, postal and email addresses, and permission to post, text, telephone or email them, as a significant proportion failed to meet face-to-face or complete and return the measures sent by post. Participating practitioners should also be trained to remind patients of their involvement in the study when they attend review appointments. It is possible that some of the apparent benefit of the intervention was due to the extra attention patients received, so it is important to have similar follow-up rates in the two arms.

Current demands on practices, and the expansion of less than full-time working, make it increasingly difficult to provide continuity of care, which may explain why participating patients sometimes found it difficult to get follow-up appointments with the same GP. Therefore, it will be important to recruit practices where all GPs and PNs in the practice agree to be involved in the study and to be trained in recruiting, consenting and following up all eligible patients, and looking at the results of PROMs for all those in the intervention arm. In a definitive trial, practices should be cluster randomised to streamline recruitment and follow-up, so all patients in each are treated the same, by whichever GP or PN they see.

Cluster randomisation tends to require a larger sample due to clustering by practice (the design effect), although the increase in sample size necessary appears likely to be small based on this study. There was no evidence of clustering at baseline, but the study may not have been large enough to permit an accurate estimation of the true value of the ICC and it would be sensible in a larger trial to make an allowance for clustering. After controlling for baseline and randomisation group, the ICC for the BDI-II at 12 weeks was 0.03. The ICC for the BDI-II from a previous trial of antidepressants for mild to moderate depression in primary care was 0.02;^31^ therefore, an ICC of 0.03 might be appropriate to use to calculate the design effect if the definitive trial is cluster randomised. This is a relatively small ICC, but if a smaller number of practices each recruiting more patients is recruited, the design effect will be greater as it increases with increasing cluster size.

Practice logs and recruitment rates in the better recruiting practices show the numbers of eligible patients per group practice will allow for more than six patients to be recruited per year, given greater commitment. Having a relatively smaller number of practices recruit more patients each will be more efficient in terms of travel to practices by the research team, and allow greater contact to be maintained with participating staff in each, to optimise practice commitment.

Administration of PROMs needs to be streamlined, and GPs provided with more guidance on how to assess the results, to avoid disappointing patients by not using the PROMs to inform care. Patients may benefit from being provided with a record of their PROM scores so they can monitor their progress.

The study team needs to spend more time at participating practices training them in the recruitment process and assisting them with setting up database searches. Practices should complete a trial recruiting period to assess their commitment, and practice research costs should be reimbursed on a per-patient/per-mail-out basis rather than paying them a lump sum at the beginning of the trial, to incentivise recruitment.

### Conclusions

Even in this small sample, the findings suggest that the use of PROMs may be beneficial in the short term, although maybe not in the longer term. It provides support for our plan to take forward a larger, definitive trial. Before we proceed however, we need to do some more work with potential participants, to identify the most promising PROM. Given that some practitioners found them time consuming and wanted more guidance on how to take account of the results in their treatment decisions, encouraging more practitioners to use PROMs requires identifying relatively brief measures which can potentially change management.
