# Supplementary material for: Patient-reported outcome measures for monitoring primary care patients with depression: PROMDEP feasibility randomised trial
Source: BMJ Open. 2017 Mar 30;7(3):e015266. doi: 10.1136/bmjopen-2016-015266 (PMC5387943; doi:10.1136/bmjopen-2016-015266)
Supplement: supplementary appendix [file bmjopen-2016-015266supp_appendix1.pdf]

Appendix 1: List of read codes used by GP practices to search records for potential participants

Read code	Read term
E2B..00	Depressive disorder NEC
1BT..11	Low mood
E200300	Anxiety with depression
Eu32z11	[X]Depression NOS
1B17.00	Depressed
1465	H/O: depression
1B17.11	C/O - feeling depressed
Eu32.00	[X]Depressive episode
E204.00	Neurotic depression reactive type
1BT..00	Depressed mood
1B1U.00	Symptoms of depression
E204.11	Postnatal depression
2257	O/E - depressed
Eu32100	[X]Moderate depressive episode
E113.11	Endogenous depression - recurrent
Eu32z00	[X]Depressive episode, unspecified
1BO..00	Mood swings
Eu32z14	[X] Reactive depression NOS
E112.13	Endogenous depression first episode
E112.14	Endogenous depression
1B1J.11	Emotional upset
E2B1.00	Chronic depression
E112.11	Agitated depression
E112.00	Single major depressive episode
E135.00	Agitated depression
E113700	Recurrent depression
Eu32000	[X]Mild depressive episode
Eu33.00	[X]Recurrent depressive disorder
Eu41200	[X]Mixed anxiety and depressive disorder
Eu32200	[X]Severe depressive episode without psychotic symptoms
1B1U.11	Depressive symptoms
E113.00	Recurrent major depressive episode
Eu32z12	[X]Depressive disorder NOS
Eu3..00	[X]Mood - affective disorders
E112.12	Endogenous depression first episode
Eu32400	[X]Mild depression
Eu32.11	[X]Single episode of depressive reaction
E113200	Recurrent major depressive episodes, moderate
Eu34100	[X]Dysthymia
E112200	Single major depressive episode, moderate
Eu32.13	[X]Single episode of reactive depression
E112100	Single major depressive episode, mild
Eu33100	[X]Recurrent depressive disorder, current episode moderate
Eu34114	[X]Persistant anxiety depression
Eu41211	[X]Mild anxiety depression
E290.00	Brief depressive reaction
Eu53011	[X]Postnatal depression NOS

Eu33.13	[X]Recurrent episodes of reactive depression
1BQ..00	Loss of capacity for enjoyment
E11z200	Masked depression
Eu33z00	[X]Recurrent depressive disorder, unspecified
Eu34113	[X]Neurotic depression
Eu33.11	[X]Recurrent episodes of depressive reaction
E112z00	Single major depressive episode NOS
E291.00	Prolonged depressive reaction
Eu43012	[X]Acute reaction to stress
Eu32y00	[X]Other depressive episodes
E113z00	Recurrent major depressive episode NOS
Eu43z00	[X]Reaction to severe stress, unspecified
E112300	Single major depressive episode, severe, without psychosis
1BT..12	Sad mood
Eu33200	[X]Recurr depress disorder cur epi severe without psyc sympt
Eu33000	[X]Recurrent depressive disorder, current episode mild
1JJ..00	Suspected depression
E113100	Recurrent major depressive episodes, mild
Eu32212	[X]Single episode major depression w'out psychotic symptoms
Eu34111	[X]Depressive neurosis
Eu32700	[X]Major depression, severe without psychotic symptoms
E113300	Recurrent major depressive episodes, severe, no psychosis
E112000	Single major depressive episode, unspecified
Eu32600	[X]Major depression, moderately severe
Eu33211	[X]Endogenous depression without psychotic symptoms
1BP0.00	Loss of interest in previously enjoyable activity
Eu33.12	[X]Recurrent episodes of psychogenic depression
Eu4..00	[X]Neurotic, stress - related and somoform disorders
E113600	Recurrent major depressive episodes, in full remission
Eu33400	[X]Recurrent depressive disorder, currently in remission
Eu3z.00	[X]Unspecified mood affective disorder
Eu32.12	[X]Single episode of psychogenic depression
Eu32z13	[X]Prolonged single episode of reactive depression
1BU..00	Loss of hope for the future
E113000	Recurrent major depressive episodes, unspecified
Eu32500	[X]Major depression, mild
Eu32y11	[X]Atypical depression
E112500	Single major depressive episode, partial or unspec remission
Eu33212	[X]Major depression, recurrent without psychotic symptoms
Eu53012	[X]Postpartum depression NOS
1S40.00	Dysphoric mood
E284.00	Stress reaction causing mixed disturbance of emotion/conduct
ZV11100	[V]Personal history of affective disorder
E113500	Recurrent major depressive episodes,partial/unspec remission
E11y200	Atypical depressive disorder
Eu3y111	[X]Recurrent brief depressive episodes
Eu33y00	[X]Other recurrent depressive disorders
Eu43y00	[X]Other reactions to severe stress
E112600	Single major depressive episode, in full remission
Eu32211	[X]Single episode agitated depressn w'out psychotic symptoms

Eu34.00	[X]Persistent mood affective disorders
E290z00	Brief depressive reaction NOS
E292.00	Adjustment reaction, predominant disturbance other emotions
E283z00	Other acute stress reaction NOS
Eu92.11	[X]Emotional behavioural problems
Eu3y.00	[X]Other mood affective disorders
Eu3y000	[X]Other single mood affective disorders
E292400	Adjustment reaction with anxious mood
Eu32y12	[X]Single episode of masked depression NOS
Eu3y100	[X]Other recurrent mood affective disorders
E292z00	Adjustment reaction with disturbance of other emotion NOS
Eu34z00	[X]Persistent mood affective disorder, unspecified
E2C4z00	Mixed disturbance of conduct and emotion NOS
Eu32213	[X]Single episode vital depression w'out psychotic symptoms
Eu33z11	[X]Monopolar depression NOS
Eu3yy00	[X]Other specified mood affective disorders
Eu32B00	[X]Antenatal depression
Eu33214	[X]Vital depression, recurrent without psychotic symptoms
Eu34y00	[X]Other persistent mood affective disorders
