# Supplementary material for: Patient-reported outcome measures for monitoring primary care patients with depression: PROMDEP feasibility randomised trial
Source: BMJ Open. 2017 Mar 30;7(3):e015266. doi: 10.1136/bmjopen-2016-015266 (PMC5387943; doi:10.1136/bmjopen-2016-015266)
Supplement: supplementary appendix [file bmjopen-2016-015266supp_appendix2.pdf]

Appendix 2: Advice given to GPs about the meaning of PROM scores

PHQ-9 Scores and Proposed Treatment Actions

(Adapted from Kroenke K, Spitzer RL, Psychiatric Annals 2002;32:509-521)

PHQ-9 scores at diagnosis

PHQ-9 Score	Depression Severity	Proposed Treatment Actions
0 – 4	None-minimal	None
5 – 9	Mild	Watchful waiting; repeat PHQ-9 at follow-up
10 – 14*	Moderate	Psychological Treatment and follow-up/Antidepressants
15 – 19	Moderately Severe	Antidepressants and Psychological Treatment
20 – 27	Severe	Referral to mental health

* Question B must be answered positively, i.e. the symptoms have made it at least somewhat difficult for the patient to do their work, take care of their home, or get along with other people.

Changes in PHQ-9 Scores at Follow-up and Proposed Treatment Actions

Change in PHQ-9 score	Response	Treatment actions
Drop of 5 or more points from baseline	Adequate	No treatment change needed. Continue treatment and follow-up as planned.
Drop of 2-4 points from baseline.	Probably Inadequate	Often warrants an increase in antidepressant dose; follow-up as planned
Drop of 1-point, or no change, or increase in score, first follow-up	Inadequate	Increase dose or switch drug<br>Refer for psychological treatment<br>Follow-up sooner
Drop of 1-point, or no change, or increase in score, subsequent follow-up	Inadequate	Increase dose or switch drug<br>Refer for psychological treatment<br>Refer to mental health

Distress Thermometer

The patient marks the thermometer to indicate how much distress they have that week, from 0 to 10. Scores above 4 indicate significant distress needing further exploration and possible action.

PSYCHLOPS

All of the responses in PSYCHLOPS are scored on a six point scale ranging from zero to five. A score of zero indicates the least psychological difficulty whereas a score of five indicates the most psychological difficulty.

The questions which are scored are those relating to Problems (Questions 1b and 2b), Functioning (Question 3b) and Wellbeing (Question 4). Other questions provide useful information but do not contribute to the total score or change score.

The initial score for PSYCHLOPS is unique to each individual. The main purpose of the score is to measure within-person change, i.e. the change in score for the items chosen by the patient.

High scores at diagnosis indicate significant problem areas for the individual patient, and should direct questioning to those areas. Similarly, persistently high scores, or increased scores, at follow-up should direct questioning to those areas of particular concern to the individual patient.
