# Supplementary figures and images for: Patient-reported outcome measures for monitoring primary care patients with depression: PROMDEP feasibility randomised trial
Source: BMJ Open. 2017 Mar 30;7(3):e015266. doi: 10.1136/bmjopen-2016-015266 (PMC5387943; doi:10.1136/bmjopen-2016-015266)

### Appendix 3: Study intervention and control procedures

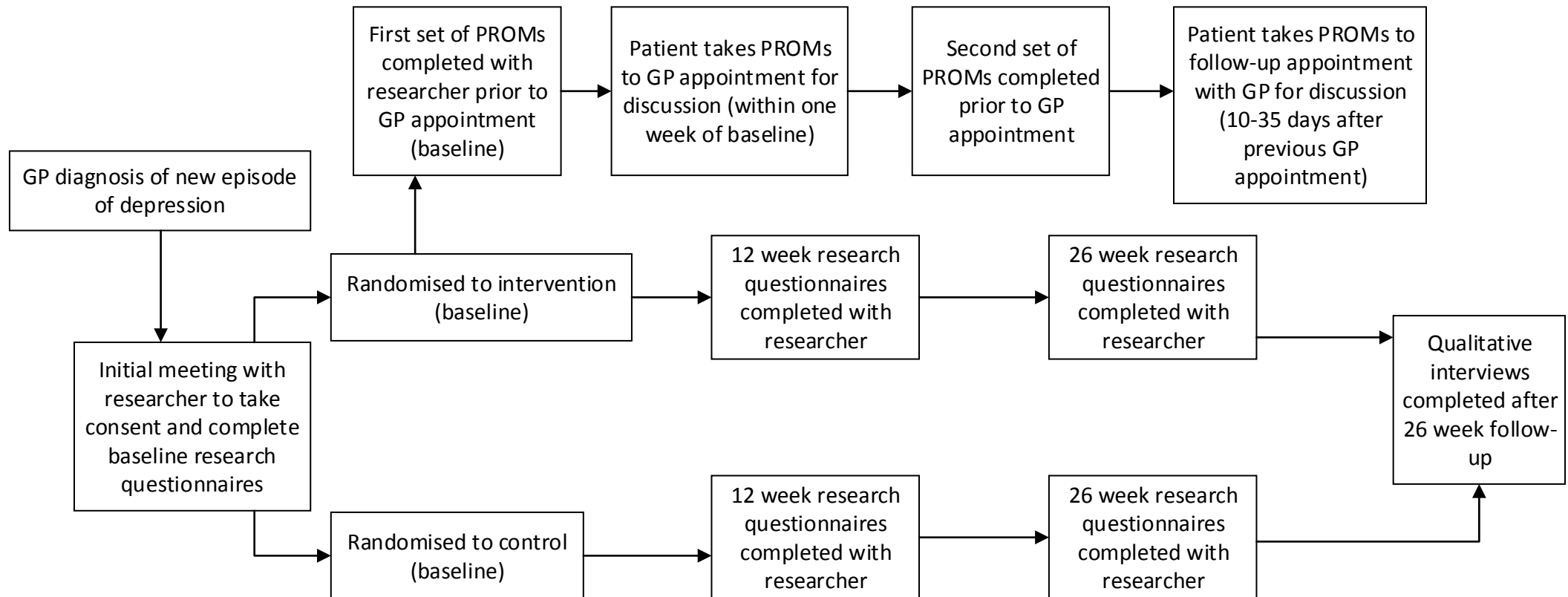

Supplement: supplementary appendix [file bmjopen-2016-015266supp_appendix3.pdf]
